# Randomized controlled clinical trial of the impact of combined application of rhamnosus probiotics and oral nystatin on the colonization of intestinal Candida albicans in low birth weight neonates <2,500 grams

**DOI:** 10.3205/dgkh000632

**Published:** 2026-02-25

**Authors:** Mazyar Vakiliamini, Reza Habibi, Samira Zarei, Roya Chegene Lorestani, Fatemeh Rezaeeniya, Farzad Mashreghi, Pourya Mohammadi, Mansour Rezaei, Mosayeb Rostamian, Hajar Motamed

**Affiliations:** 1 Clinical Research Development Center, Imam Khomeini and Mohammad Kermanshahi and Farabi Hospitals, Kermanshah University of Medical Sciences, Kermanshah, Iran; 2 Department of Microbiology, School of Sciences, Kherad Institute of Higher Education, Bushehr, Iran; 3 Infectious Diseases Research Center, Health Policy and Promotion Institute, Kermanshah University of Medica Sciences, Kermanshah, Iran; 4 School of Medicine, Lorestan University of Medical Sciences, Khorramabad, Iran; 5 Department of Parasitology, School of Medicine, Qazvin University of Medical Sciences, Gazvin, Iran; 6 Department of Biostatistics, School of Health, Kermanshah University of Medical Sciences, Kermanshah, Iran; 7 Department of Pathology, School of Medicine, Kermanshah University of Medical Sciences Kermanshah, Iran

**Keywords:** Candida albicans, enteral colonization, neonates, Rhamnosus probiotics, oral nystatin, shortened hospital stay, improved food intake, fewer NPO days

## Abstract

**Objectives:**

This study aimed to evaluate the concurrent effects of consuming Rhamnosus probiotics and oral antifungal Nystatin on inhibiting *Candida (C.) albicans* colonization in infants weighing <2,500 grams.

**Materials:**

This study was a randomized controlled clinical trial conducted on 104 infants born weighing <2,500 grams at Mohammad Kermanshahi Hospital in Kermanshah in 2021–22. The infants were randomly allocated into intervention (n=52) and control (n=52) groups. The control group received a placebo, 10 drops orally four times daily. In contrast, the intervention group received oral Nystatin 100,000 units 10 drops three times a day, combined with Rhamnosus Lactobacillus oral drops once daily for 7 to 10 days.

**Results:**

The results showed a lower frequency of positive *C. albicans* cultures in stool samples on the 7^th^ day of age in the intervention group. Moreover, several variables such as the onset of nutrition, time to reach 100 ml/kg of milk intake per day, number of nil per os (NPO; “nothing through the mouth”) episodes, total NPO time during hospitalization, episodes of intolerance until discharge, and duration of hospitalization were significantly lower in the intervention group compared to the control.

**Discussion:**

The concurrent use of Rhamnosus probiotics and oral Nystatin can lead to a reduction of positive *C. albicans* stool culture results, average time for certain variables such as the onset of nutrition, time to reach 100 ml/kg of milk intake per day, NPO days, total NPO time during hospitalization, episodes of intolerance until discharge, and duration of hospitalization in infants weighing <2,500 grams. Hence, their simultaneous administration is recommended for these neonates.

## Zusammenfassung

**Zielsetzung:**

Es sollte die Auswirkung der gleichzeitigen Einnahme von Rhamnosus-Probiotika und dem oralen Antimykotikum Nystatin auf die Hemmung der Besiedlung mit *Candida (C.) albicans* bei Säuglingen mit einem Gewicht <2.500 Gramm untersucht werden.

**Methode:**

Die randomisierte, kontrollierte, klinische Studie wurde bei 104 Neonaten mit einem Geburtsgewicht <2,500 g im Mohammad Kermanshahi Hospital in Kermanshah in den Jahren 2021–22 durchgeführt. Die Neonaten wurden nach dem Zufallsprinzip in eine Interventionsgruppe (n=52) und eine Kontrollgruppe (n=52) eingeteilt. Die Kontrollgruppe erhielt viermal täglich 10 Tropfen eines Placebos oral. Die Interventionsgruppe erhielt dagegen dreimal täglich 10 Tropfen Nystatin 100.000 Einheiten oral, kombiniert mit einmal täglich Rhamnosus Lactobacillus-Tropfen oral über einen Zeitraum von 7 bis 10 d.

**Ergebnisse:**

Am 7. Lebenstag zeigte sich in der Interventionsgruppe eine geringere Häufigkeit positiver *C.-albicans*-Kulturen in Stuhlproben. Darüber hinaus waren mehrere Variablen wie der Zeitpunkt des Beginns der Ernährung, die Zeit bis zum Erreichen einer Milchaufnahme von 100 ml/kg/d, die Anzahl der nil per os (NPO)-Episoden, die Gesamt-NPO-Zeit während des Krankenhausaufenthalts, die Episoden von Unverträglichkeit bis zur Entlassung und die Dauer des Krankenhausaufenthalts in der Interventionsgruppe im Vergleich zur Kontrollgruppe signifikant geringer ausgeprägt.

**Diskussion:**

Die gleichzeitige Anwendung von Rhamnosus-Probiotika und oralem Nystatin kann bei Säuglingen mit einem Gewicht <2.500 g zu einer Verringerung der positiven Stuhlkulturergebnisse mit Nachweis von *C. albicans* und der Variablen Beginn der Ernährung, Zeit bis zum Erreichen einer Milchaufnahme von 100 ml/kg/d, Anzahl Tage ohne orale Nahrungsaufnahme (NPO), Gesamtdauer der NPO während des Krankenhausaufenthalts, Episoden von Unverträglichkeit bis zur Entlassung und Dauer des Krankenhausaufenthalts führen. Daher wird die gleichzeitige Verabreichung beider Mittel für Neonaten mit einem Gewicht <2.500 g empfohlen.

## Introduction

In recent years, the incidence of invasive fungal infections has notably increased in surviving low-birth-weight infants, leading to a significant mortality rate of 30% [1]. Premature and low birth weight neonates are particularly vulnerable due to their immature defense systems, delicate skin structure, fragile gastrointestinal mucosa, invasive diagnostic and treatment procedures, broad-spectrum antibiotic prescriptions, and the moist hospital environment, resulting in invasive candidiasis from intestinal colonization [2]. 

This colonization occurs via *Candida* strains transferred vertically from mothers or horizontally from healthcare workers or the hospital environment [3]. Reports indicate that 7 to 20% of premature neonates are colonized with *Candida* spp., predisposing them to invasive candidiasis [4] ,[5], [6], [7]. This infection intensifies with decreasing age and weight, leading to a higher prevalence of invasive fungal infections (IFIs) in very low birth weight infants (<1,500 grams) at birth, estimated at 1–9%, and in extremely low birth weight infants (<1,000 grams) at 15% [2], [8], [9], [10]. IFIs significantly impact the survival of preterm infants with prolonged hospitalization [2], [11], [12].

Moreover, early diagnosis of the infection remains challenging, resulting in treatment delays. Therefore, employing antifungal agents as a preventive measure against invasive fungal infections in this population is common. Preventing *Candida* ssp. intestinal colonization or treating existing cases can reduce the occurrence of necrotizing enterocolitis (NEC) or invasive candidiasis, increasing survival chances and reducing morbidity and mortality rates. Proposed methods for prophylaxis include prescribing intravenous fluconazole to infants with low birth weight or those with central venous catheters [13], the use of probiotics [14], [15], [16], the administration of oral synbiotic in low birth weight infants to prevent intestinal colonization [17], [18], and the utilization of oral antifungals such as nystatin and oral fluconazole for prophylaxis and treatment of intestinal colonization in preterm and low birth weight infants [19], [20], [21], [22], [23].

Previous investigations have addressed the individual roles of probiotic *Lactobacillus (L.) rhamnosus* and oral nystatin; however, the effectiveness of these two medications used in conjunction has not been assessed. Hence, this study aimed to examine the combined effect of these two drugs on preventing *C. albicans* intestinal colonization.

## Materials and methods

This study was a comparative randomized clinical trial utilizing a triple-blind approach. In this clinical trial, the triple-blinding involved the sample groups (intervention and control), researchers, treatment teams, or any individuals involved with the samples (including statistical experts) responsible for data analysis, being unaware of the specific interventions or treatments. The study population consisted of neonates weighing <2,500 grams from June 30, 2021, to November 10, 2021, at Dr. Mohammad Kermanshahi Educational Hospital. Inclusion criteria involved birth weight <2,500 grams, hospitalization age of 72 hours or less, mothers not using oral or topical antifungals in the final months of pregnancy, and no consumption of artificial milk containing prebiotics or probiotics. Infants were excluded if they died within 7 days of birth due to congenital anomalies of the oral cavity or gastrointestinal tract, sepsis, simultaneous use of oral and injectable antifungals, or severe hemodynamic disorders.

For random allocation, a permuted randomization block method was employed to ensure balance in allocating participants into each study group. This method involved creating blocks of four (two participants in the intervention group and two in the control group). Sampling continued until the completion of the required sample size. In executing the randomization process, the individual responsible for the randomization schedule was separated from other researchers to minimize selection bias. A blinding strategy was implemented to prevent bias during and after the intervention. This entailed participants and outcome assessors being unaware of the nature of the control or intervention of groups A and B and the differences in interventions. Thus, the study was triple-blind.

According to the inclusion or exclusion criteria, 104 newborns weighing <2,500 grams were randomly assigned (for homogeneity) into two groups of 52 each. The age of these infants ranged from 1 to 3 days. The drug administration period lasted from 7 to 10 days. The control group received four doses of 10 drops of placebo (distilled water) daily. In comparison, the intervention group received three doses daily of 10 drops of oral nystatin (100,000 units) and one daily dose of 10 drops of oral *L. rhamnosus*, which contains 5×10^5^ active lyophilized cells of *L. rhamnosus* per 10 drops. In Iran, this product is a 5-milliliter droplet stored at 2 to 8°C. No potential side effects, toxicity, or drug interactions have been reported. Two specialized *C. albicans* cultures were taken from each infant’s feces. The first sample was collected between 1 to 3 days of birth, before the commencement of medications, and the second was collected after 7 days. All fecal samples were promptly transferred to the laboratory and were processed within two hours of collection in a general medium, such as Sabouraud dextrose agar. For* C. albicans*, 0.1 gram of feces was diluted in 1.0 mL normal saline, and then 10 µL were cultured in a petri dish containing Sabouraud dextrose agar. The cultured petri dishes were incubated at 35°C for 48 hours, followed by yeast identification using germ tubes to confirm the presence of *C. albicans*. 

The sample collection and culturing were conducted blindly, and measures were taken to prevent bias in culturing results. Following the receipt of results, comparisons were made between the infants in terms of age, gender, weight, mode of delivery, occurrence of systemic instability symptoms, NEC, intolerance, initiation of feeding, time to reach maximum feeding, frequency of nil per os (NPO) occurrences due to intolerance, duration of hospitalization, etc.

Data analysis in this study was performed using SPSS version 24 software. If necessary, the chi-squared test or Fisher’s exact test was utilized for qualitative variable comparisons. For before and after comparisons within each group, the paired t-test was used. The Kolmogorov-Smirnov (KS) test assessed normality in quantitative data. For normally distributed quantitative data, independent t-tests were employed; for non-normally distributed data, the Mann-Whitney U-test was utilized. Covariate control was implemented using analysis of covariance (ANCOVA) or categorical methods as needed for 18 confounding variables. The intention-to-treat (ITT) analysis was applied in analyzing this trial. Results were deemed statistically significant if p<0.05.

This study was registered in the Iranian Registry of Clinical Trials (IRCT registration code: IRCT 20130812014333N175). Moreover, written informed consent forms were obtained from the neonates’ parents.

The protocol was approved by the Ethics Committee of Kermanshah University of Medical Sciences (IR.KUMS.REC.1400.426).

## Results

Out of 104 infants examined, in the intervention group, 26 (50%) were male and 26 (50%) were female, while in the control group, 30 (57%) were male and 22 (42%) were female.

There were no statistically significant differences between the intervention and control groups regarding gender, mode of delivery, distention, vomiting, occurrence of NEC, clinical signs, suspected and proven NEC cases, mortality within 7 days, gestational age, birth weight, weight at admission, and weight at discharge (Table 1 (Tab. 1)).

The results of culturing *C. albicans* from stool samples in infants aged 1 to 3 days before intervention in the intervention and control groups were 4 (7.7%) and 11 (21.2%), respectively, indicating a noticeable but not statistically significant decrease in the intervention group.

The number of positive cultures at 7 days of age in the intervention and control groups was 0 (0%) and 9 (17.3%), respectively. There was a statistically significant difference between the intervention and control groups in culturing *C. albicans* from stool samples at 7 days of age, indicating a significantly higher rate of positive cultures in the control group than in the intervention group (Table 1 (Tab. 1)).

The intervention and control groups significantly differed in the frequency of reaching milk intake at 100 mL/kg, with a higher percentage of children in the intervention group initiating complementary feeding before 3 days than the control group.

The frequency of zero occurrences for NPO, the total duration of NPO, and the frequency of zero occurrences for intolerance were significantly higher in the intervention group compared to the control group.

There was a statistically significant difference in the length of hospital stay until discharge (in days) between the two groups, with the intervention group showing significantly fewer days spent in the hospital until discharge than the control group.

**Figure 1:**
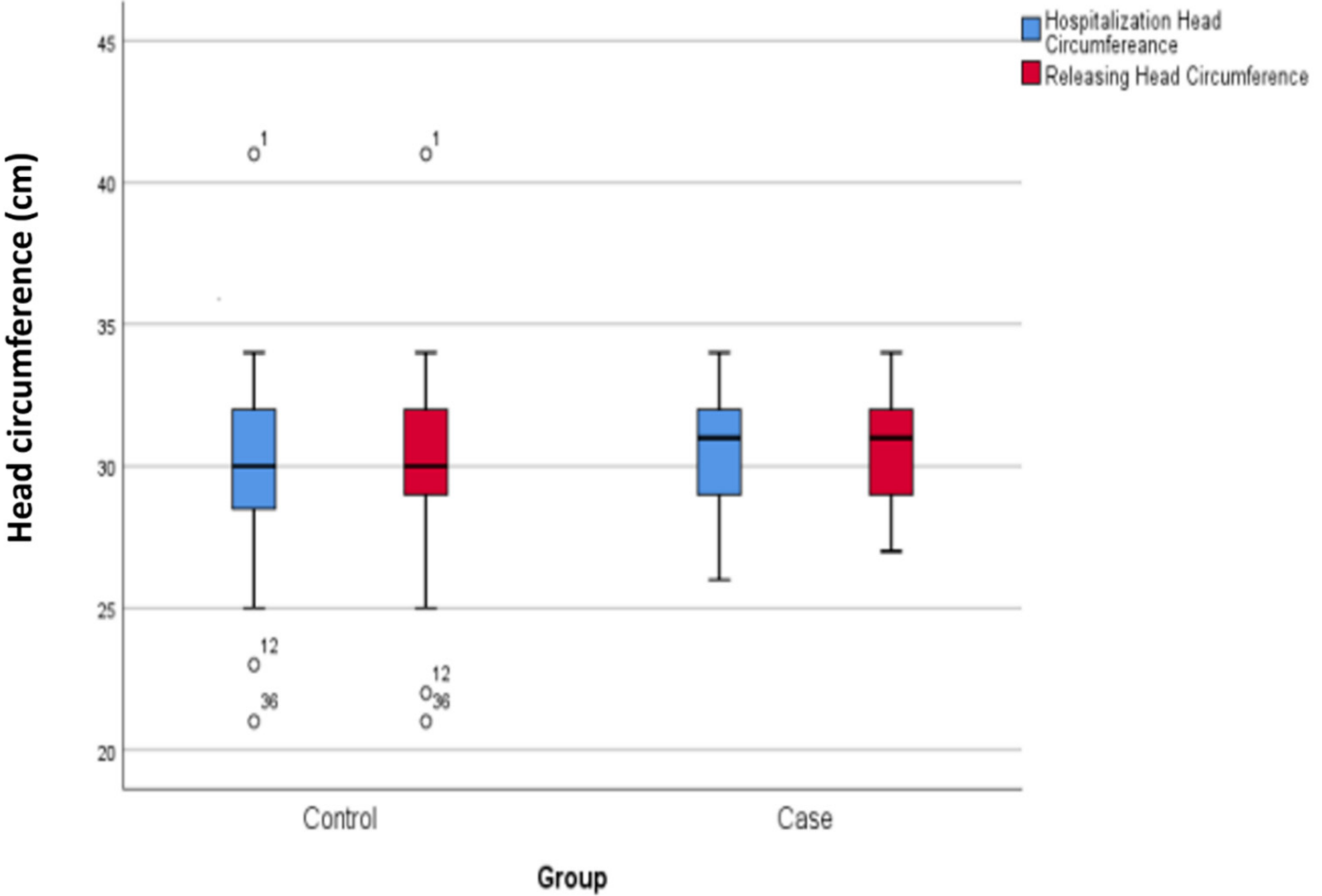
Mean head circumference during hospitalization and at discharge in the case and control groups

At admission, the mean head circumference of the two groups (intervention group: 30.56±1.83, control group: 30.20±2.95) did not show a statistically significant difference. Similarly, the mean head circumference of the two groups (intervention group: 30.65±1.77, control group: 30.32±2.92) at discharge did not demonstrate a statistically significant difference (Figure 1 (Fig. 1)).

## Discussion

*C. albicans* is a significant hospital-acquired pathogen leading to complications and mortality in premature and low birth weight infants [24]. The findings of this study revealed a substantial decrease in positive *C. albicans* cultures from stool samples at 7 days of age and a significant difference in mean variables, including the time until the onset of feeding, time to reach 100 mL/kg of milk intake per day, number of NPO episodes, cumulative NPO time during hospitalization, number of intolerance episodes until discharge, and length of hospital stay between the intervention and control group. However, there were no significant differences between the intervention and control groups concerning *C. albicans* cultures at 1 to 3 days of age, the occurrence of distension, vomiting, NEC symptoms, clinical signs, mortality within 7 days, mean age at admission, discharge weight, and length of stay at discharge.

Our study aligns with previous research in this field. Various studies have indicated that probiotics may reduce intestinal colonization in low-weight infants [16], [17], [18]. Another study reported that using *L. reuteri and L. rhamnosus* probiotics can prevent gastrointestinal *Candida* spp colonization, protect against late-onset sepsis, and reduce neurodevelopmental abnormalities in premature infants [25]. A comparative study of probiotics with nystatin demonstrated that *L. reuteri* is as effective as nystatin in reducing *Candida* colonization and might serve as a safe and effective alternative to anti-yeast drugs for low-birth-weight infants [26]. Studies have shown that probiotics can reduce *Candida* colonization and invasive infections due to *C. albicans* in rodent models [27] ,[28], [29].

Some other studies have proposed using oral anti-yeast agents such as nystatin and fluconazole for prophylaxis and treating intestinal colonization with *Candida* species in premature and low-weight neonates [19], [20], [21], [22]. In a randomized double-blind clinical trial, Manzoni et al. [30] reported that oral administration of *L. rhamnosus* and *L. reuteri* reduces the incidence and severity of *Candida* gut colonization without adverse effects. These researchers suggested that using probiotics and fluconazole to prevent fungal colonization in infants weighing <1,000 grams at birth could lead to a reduction in intestinal colonization with Candida spp.

In a systematic review conducted in 2018, 51 articles were reviewed. Among various probiotic combinations, 25 articles indicated a reduction in mortality. Seven articles confirmed a decrease in NEC occurrence. In comparison, two articles confirmed a reduction in length of stay, and three articles endorsed a decrease in the time taken for complete feeding. Provided all safety issues have been addressed, there is currently a conditional recommendation (with low certainty of evidence) to provide either *L. rhamnosus* GG ATCC53103 or the combination of *Bifidobacterium (B.) infantis*, *B. lactis* and *Streptococcus thermophilus* to reduce NEC rates [31].

Our findings also demonstrate that simultaneous use of *L. rhamnosus* probiotic and oral antifungal nystatin can lead to a decrease in positive *C. albicans* stool culture at 7 days of age and significant differences in mean variables such as the time to initiate feeding, time to reach 100 mL/kg of milk intake per day, the number of NPO episodes, cumulative NPO time during hospitalization, number of intolerance episodes until discharge, and length of hospital stay until discharge. Therefore, their usage is recommended in neonates weighing less than 2,500 grams.

## Conclusions

Based on the results of the studies mentioned above, probiotics can be considered an alternative preventive and therapeutic approach. In essence, probiotics competitively exclude *Candida* spp., stimulate the host immune system, inhibit epithelial and mucosal adhesion, and restrain epithelial invasion [32], [33].

## Notes

### Competing interests

The authors declare that they have no competing interests.

### Ethical approval 

The protocol was approved by the Ethics Committee of Kermanshah University of Medical Sciences (IR.KUMS.REC.1400.426).

### Funding

None. 

### Acknowledgments

We thank Dr. Mohammad Kermanshahi Hospital, Kermanshah University of Medical Sciences for their support.

### Authors’ ORCIDs

- Vakiliamini M: https://orcid.org/0000-0002-9677-3846
- Habibi R: https://orcid.org/0000-0002-4579-4997
- Chegene Lorestani R: https://orcid.org/0000-0002-8137-5378
- Mohammadi P: https://orcid.org/0000-0002-4313-3255
- Rezaei M: https://orcid.org/0000-0002-6446-7289
- Rostamian M: https://orcid.org/0000-0002-1071-7019
- Motamed H: https://orcid.org/0000-0002-4419-0497

## Figures and Tables

**Table 1:**
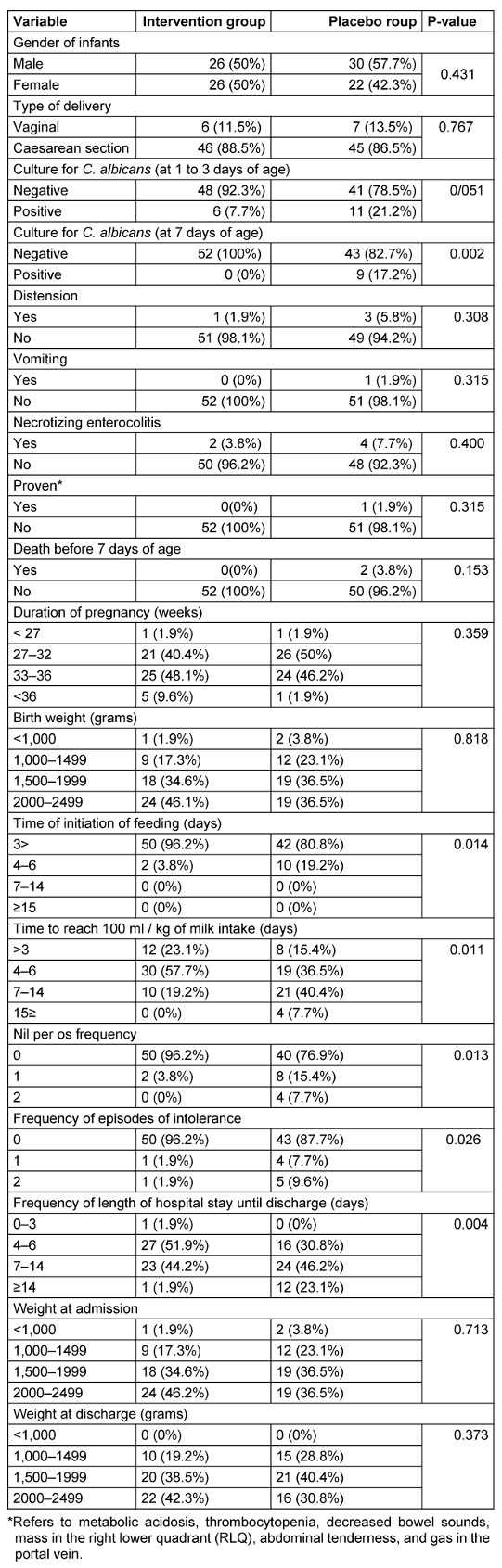
Comparison of demographic information and clinical signs between intervention group and placebo group
